# Supplementary material for: Systematic detection of functional proteoform groups from bottom-up proteomic datasets
Source: Nat Commun. 2021 Jun 21;12:3810. doi: 10.1038/s41467-021-24030-x (PMC8217233; doi:10.1038/s41467-021-24030-x)
Supplement: Supplementary file 1 — Supplementary Information [file 41467_2021_24030_MOESM1_ESM.pdf]

Supplementary information for

Systematic detection of functional proteoform groups from bottom-up proteomic datasets

Isabell Bludau[1,2], Max Frank[1,3,4,5], Christian Dörig[1], Yujia Cai[3,4], Moritz Heusel[1,6], George Rosenberger[1,7], Paola Picotti[1], Ben C. Collins[1,8], Hannes Röst[3,4,*], Ruedi Aebersold[1,9,*]

1) Department of Biology, Institute of Molecular Systems Biology, ETH Zurich, Zurich, Switzerland.
2) Department of Proteomics and Signal Transduction, Max Planck Institute of Biochemistry, Martinsried, Germany.
3) Department of Molecular Genetics, University of Toronto, Toronto, Canada
4) The Donnelly Centre for Cellular and Biomolecular Research, University of Toronto, Toronto, Canada.
5) European Molecular Biology Laboratory, Genome Biology Unit, Heidelberg, Germany.
6) Division of Infection Medicine (BMC), Department of Clinical Sciences, Lund University, Lund, Sweden.
7) Columbia University, New York, NY, USA.
8) School of Biological Sciences, Queen's University Belfast, Belfast, UK.
9) Faculty of Science, University of Zurich, Zurich, Switzerland.

*) Corresponding author. E-mail: hannes.rost@utoronto.ca, aebersold@imsb.biol.ethz.ch

Supplementary Table 1: Glossary.

Term	Definition
Proteoform	A protein species with a unique combination of amino acid sequence and post-translational modifications. Multiple alternative proteoforms can originate from the same gene locus.
Functional proteoform group	A group of peptides, derived from the same gene, that co-vary across a large and heterogeneous dataset. A proteoform group can, but does not have to resemble a unique, specific proteoform.
Protein with functional proteoform groups	A protein with at least two groups of peptides that show different patterns across a large and heterogeneous dataset.

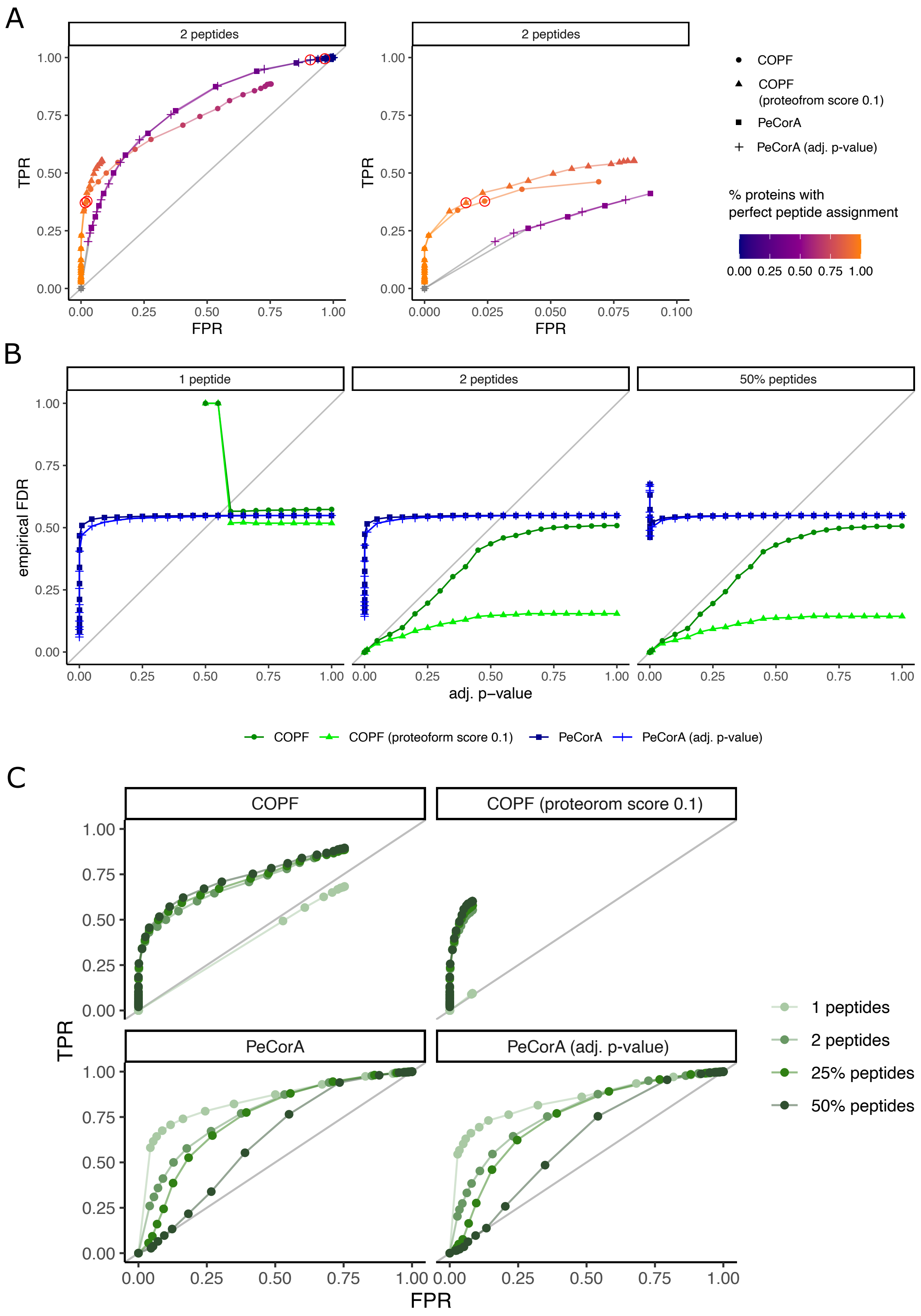

A
2 peptides
2 peptides
TPR
FPR
COPF
COPF (proteofrom score 0.1)
PeCorA
PeCorA (adj. p-value)
% proteins with perfect peptide assignment
0.00 0.25 0.50 0.75 1.00
B
1 peptide
2 peptides
50% peptides
empirical FDR
adj. p−value
COPF
COPF (proteoform score 0.1)
PeCorA
PeCorA (adj. p-value)
C
COPF
COPF (proteorom score 0.1)
PeCorA
PeCorA (adj. p-value)
TPR
FPR
1 peptides
2 peptides
25% peptides
50% peptides

Supplementary Figure 1. Additional information about the COPF and PeCorA performance benchmark. (A) Receiver operator characteristic (ROC) curves for the second in silico benchmark dataset including proteins for which proteoforms differed by two peptides. They show the true positive rate (TPR) over the false positive rate (FPR). Individual points in the curve are generated by iterating over different adjusted p-value thresholds. The datapoints derived from an adjusted p-value threshold of 0.1 are highlighted by a red circle. The right panel shows a zoom-in on the range of FPRs between 0 and 0.1. Adjusted p-values from PeCorA were derived by performing an additional multiple testing correction of the adjusted p-values reported by PeCorA. **(B)** Empirical false discovery rate (FDR) values versus estimated adjusted p-values. Individual points in the curves are generated by iterating over different adjusted p-value thresholds. **(C)** Illustration of ROC curves for COPF and PeCorA with different score thresholds for a variety of different benchmarking sets with 1, 2, 25% or 50% of peptides in an alternative proteoform.

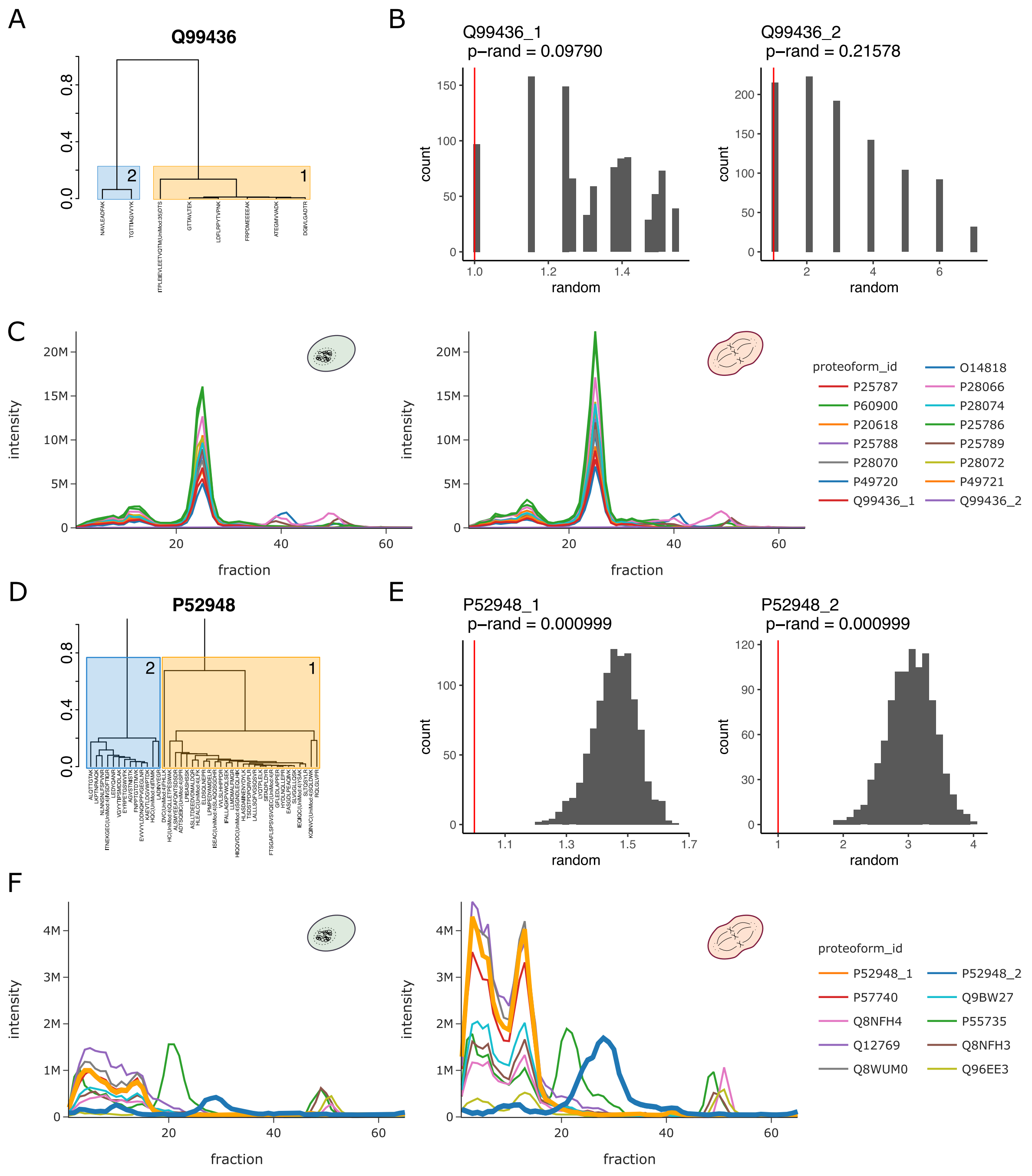

Supplementary Figure 2. Additional information about COPF results for PSMB7 and NUP98/96. (A) Clustering dendrogram for proteasome subunit beta type-7 (β7, PSMB7, UniProt ID: Q99436). The two determined proteoforms are highlighted in orange and blue. **(B)** Histograms for the sequence proximity analysis of PSMB7 proteoforms 1 (left) and 2 (right). The grey bars illustrate the proximity score distribution for 1000-times randomly shuffled peptide ranks. The red vertical line indicates the score achieved on the true, observed peptide position rank. **(C)** Protein profiles for all annotated 20S proteasome subunits in interphase (left) and mitosis (right). **(D)** Clustering dendrogram for the *Nup98* gene product (UniProt ID: P52948). The two determined proteoforms are highlighted in orange and blue. **(E)** Histograms for the sequence proximity analysis of the NUP98 proteoforms 1 (left) and 2 (right). The grey bars illustrate the proximity score distribution for 1000-times

randomly shuffled peptide ranks. The red vertical line indicates the score achieved on the true, observed peptide position rank. **(F)** Protein profiles for all annotated Nup107-160 sub-complex subunits in interphase (left) and mitosis (right).

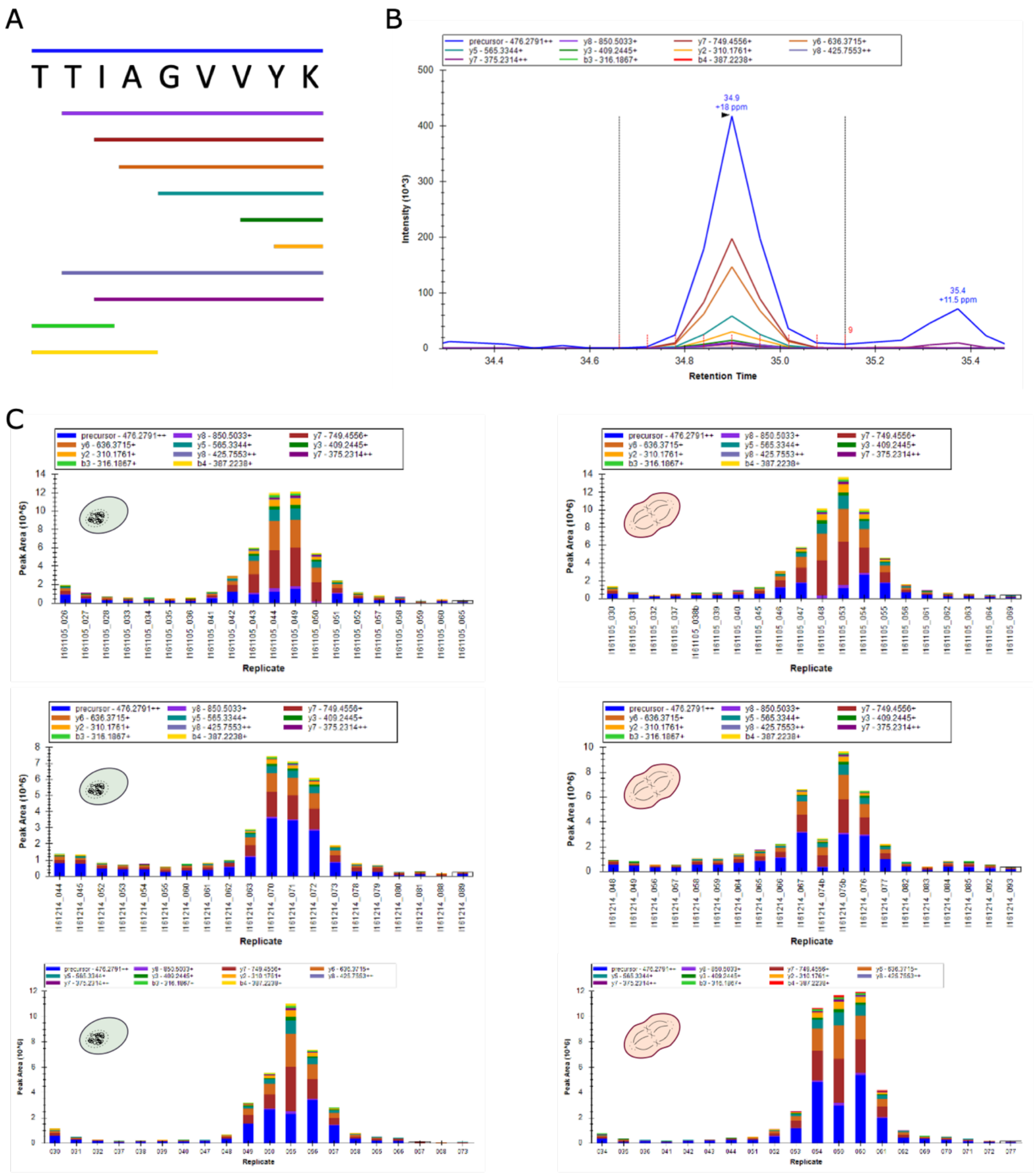

Supplementary Figure 3. Skyline extraction of the semi-tryptic peptide generated by proteolytic cleavage of the pro-peptide of PSMB7. (A) Peptide sequence of the expected semi-tryptic peptide (TTIAGVVYK) generated by proteolytic cleavage of the pro-peptide in PSMB7. The full precursor sequence is indicated in blue on top of the sequence while different fragment ions are indicated as horizontal lines below the sequence. **(B)** A representative fragment-ion peak group for TTIAGVVYK that was extracted in Skyline. **(C)** Quantitative bar plot of the extracted TTIAGVVYK signals across the three replicates in interphase (left) and mitosis (right).

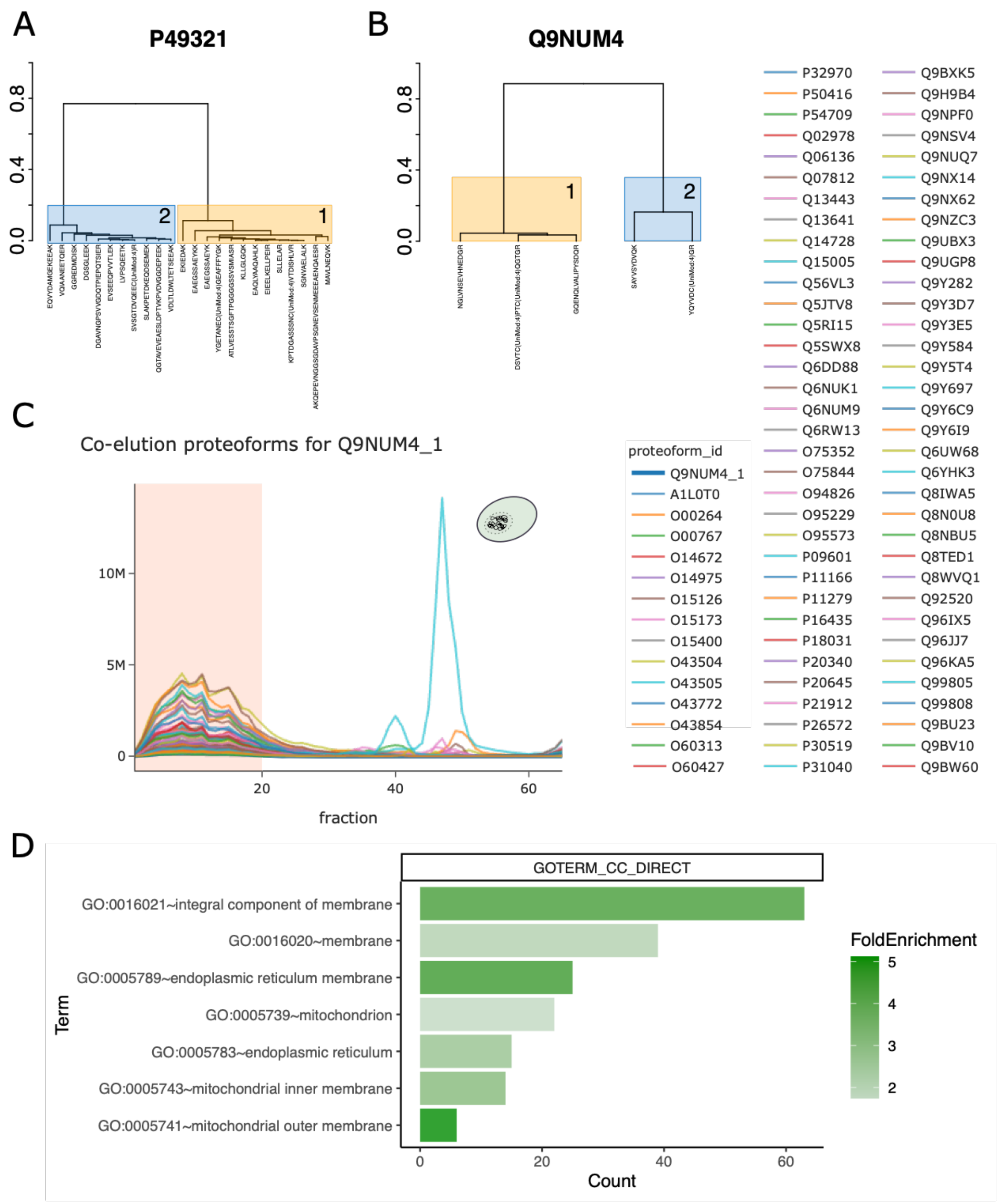

Supplementary Figure 4. Additional information on proteoform groups detected for NASP and TMEM106B. (A) Clustering dendrogram for nuclear autoantigenic sperm protein (NASP, UniProt ID: P49321). The two determined proteoforms are highlighted in orange and blue. **(B)** Clustering dendrogram for Transmembrane protein 106B (TMEM106B, UniProt ID: Q9UM4). The two determined proteoforms are highlighted in orange and blue. **(C)** High-correlating protein profiles for TMEM106B proteoform group 1 (Q9NUM4_1) in interphase. A fraction range from 1 to 20 and a minimum Pearson correlation of 0.95 were selected. **(D)** Enriched gene ontology (GO) based cellular components among the proteins that are highly correlated with TMEM106B proteoform group 1.

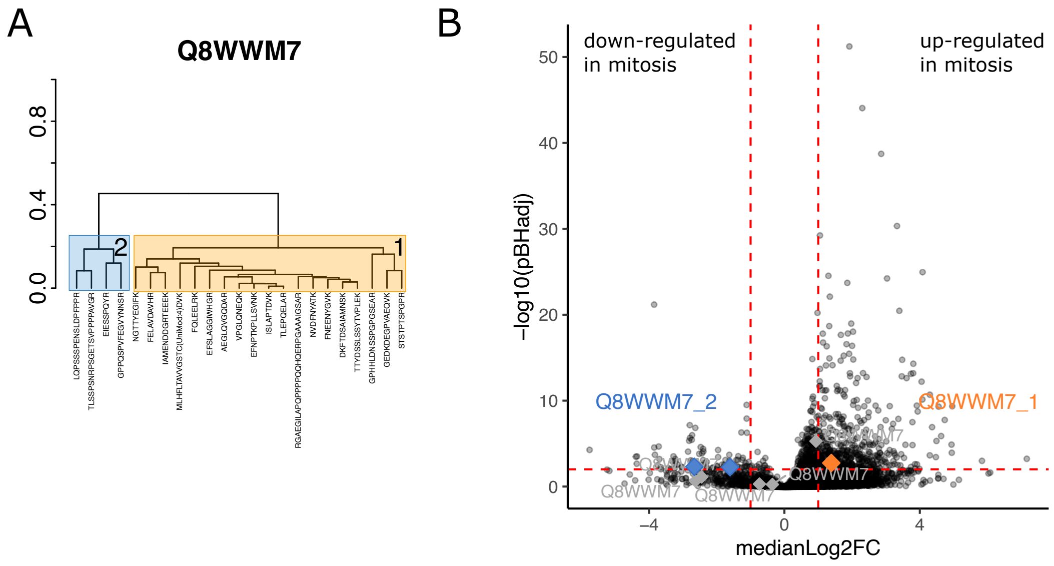

Supplementary Figure 5. Additional information on proteoform groups detected for ATXN2L. (A) Clustering dendrogram for ataxin-2-like protein (ATXN2L, UniProt ID: Q8WWM7). The two determined proteoforms are highlighted in orange and blue. **(B)** Volcano plot showing the median log-2 fold-change (medianLog2FC) over the negative Benjamini-Hochberg corrected p-value (-log10(pBHadj)). Each point represents a detected SEC elution peak for a specific proteoform group. The diamonds indicate the detected peaks for ATXN2L (UniProt ID: Q8WWM7). The colored diamonds are significantly up- or down-regulated signals (FDR ≤ 5% and absolute log-2 fold-change ≥ 1) for either of the two proteoform groups represented in orange and blue, respectively.

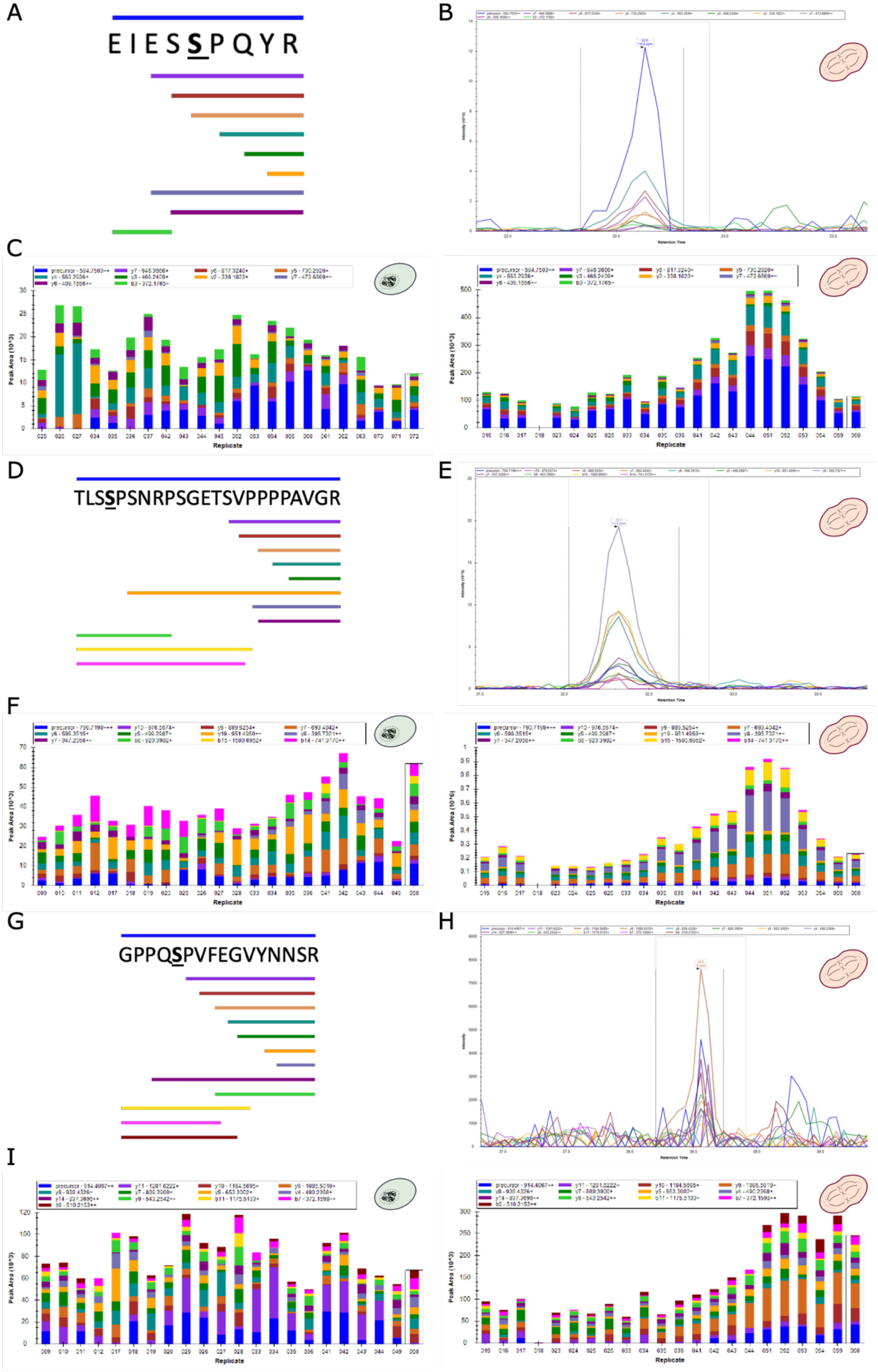
A
EIESSPQYR
B
C
Peak Area (10^3)
Replicate
D
TLSSPSNRPSGETSVPPPPAVGR
E
F
G
GPPQSPVFEGVYNNSR
H
I

Supplementary Figure 6. Skyline extraction of three phospho-peptides of ATXN2L. (A) Peptide sequence of the expected phospho-peptide EIES**S**PQYR, the expected phospho-site is highlighted in bold. The full precursor sequence is indicated in blue on top of the sequence while different fragment ions are indicated as horizontal lines below the sequence. **(B)** A representative fragment-ion peak group for EIES**S**PQYR that was extracted in Skyline (mitosis sample). **(C)** Quantitative bar plots for the extracted TLS**S**PSNRPSGETSVPPPPAVGR signals across one representative replicate in interphase (left) and mitosis (right). **(D)** Peptide sequence of the expected phospho-peptide TLS**S**PSNRPSGETSVPPPPAVGR, the expected phospho-site is highlighted in bold. The full precursor sequence is indicated in blue on top of the sequence while different fragment ions are indicated as horizontal lines below the sequence. **(E)** A representative fragment-ion peak group for TLS**S**PSNRPSGETSVPPPPAVGR that was extracted in Skyline (mitosis sample). **(F)** Quantitative bar plots for the extracted TLS**S**PSNRPSGETSVPPPPAVGR signals across one representative replicate in interphase (left) and mitosis (right). **(G)** Peptide sequence of the expected phospho-peptide GPPQ**S**PVFEGVYNNSR, the expected phospho-site is highlighted in bold. The full precursor sequence is indicated in blue on top of the sequence while different fragment ions are indicated as horizontal lines below the sequence. **(H)** A representative fragment-ion peak group for GPPQ**S**PVFEGVYNNSR that was extracted in Skyline (mitosis sample). **(I)** Quantitative bar plots for the extracted GPPQ**S**PVFEGVYNNSR signals across one representative replicate in interphase (left) and mitosis (right).

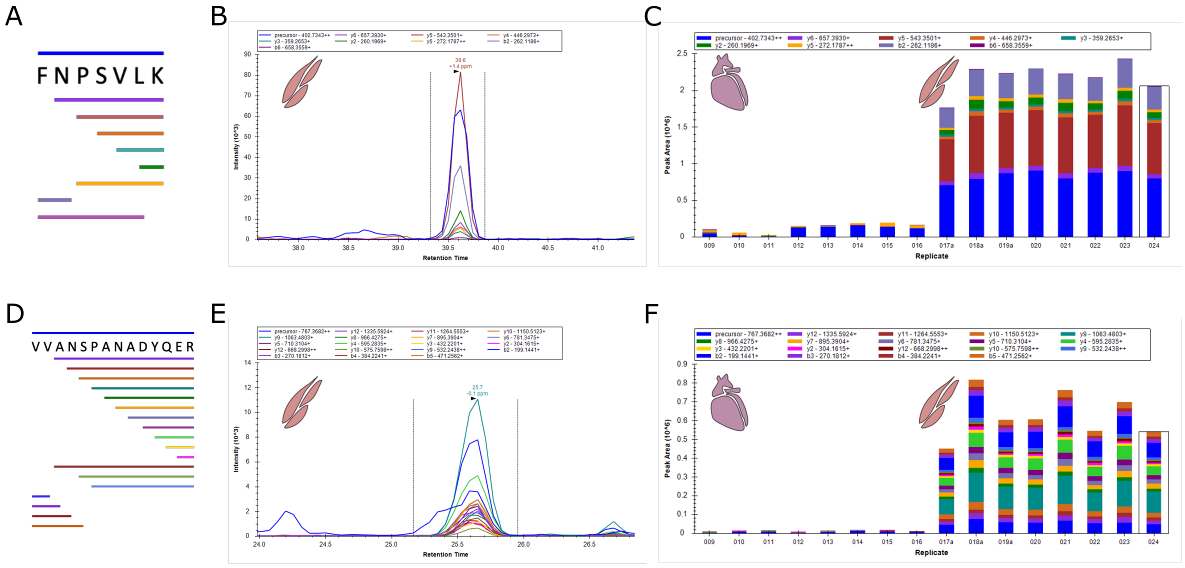

Supplementary Figure 7. Skyline extraction of peptides specific to a known skeletal muscle-specific splice isoform of Ldb3 (cypher). (A) Peptide sequence of the expected skeletal muscle-specific splice isoform FNPSVLK. The full precursor sequence is indicated in blue on top of the sequence while different fragment ions are indicated as horizontal lines below the sequence. **(B)** A representative fragment-ion peak group for FNPSVLK that was extracted in Skyline from a quadriceps sample. **(C)** Quantitative bar plots for the extracted FNPSVLK signals across the heart (left) and quadriceps samples (right). **(D)** Peptide sequence of the expected skeletal muscle-specific splice isoform VVANSPANADYQER. The full precursor sequence is indicated in blue on top of the sequence while different fragment ions are indicated as horizontal lines below the sequence. **(E)** A representative fragment-ion peak group for VVANSPANADYQER that was extracted in Skyline from a quadriceps sample. **(F)** Quantitative bar plots for the extracted VVANSPANADYQER signals across the heart (left) and quadriceps samples (right).
