# Supplementary material for: Systematic detection of functional proteoform groups from bottom-up proteomic datasets
Source: Nat Commun. 2021 Jun 21;12:3810. doi: 10.1038/s41467-021-24030-x (PMC8217233; doi:10.1038/s41467-021-24030-x)
Supplement: Supplementary file 3 — Reporting Summary [file 41467_2021_24030_MOESM3_ESM.pdf]

nature research

Corresponding author(s): Ruedi Aebersold
Hannes Roest

Last updated by author(s): May 17, 2021

Reporting Summary

Nature Research wishes to improve the reproducibility of the work that we publish. This form provides structure for consistency and transparency in reporting. For further information on Nature Research policies, see our Editorial Policies and the Editorial Policy Checklist.

Statistics

For all statistical analyses, confirm that the following items are present in the figure legend, table legend, main text, or Methods section.

n/a	Confirmed	
☐	☒	The exact sample size (*n*) for each experimental group/condition, given as a discrete number and unit of measurement
☐	☒	A statement on whether measurements were taken from distinct samples or whether the same sample was measured repeatedly
☐	☒	The statistical test(s) used AND whether they are one- or two-sided<br>*Only common tests should be described solely by name; describe more complex techniques in the Methods section.*
☐	☒	A description of all covariates tested
☐	☒	A description of any assumptions or corrections, such as tests of normality and adjustment for multiple comparisons
☐	☒	A full description of the statistical parameters including central tendency (e.g. means) or other basic estimates (e.g. regression coefficient) AND variation (e.g. standard deviation) or associated estimates of uncertainty (e.g. confidence intervals)
☐	☒	For null hypothesis testing, the test statistic (e.g. *F*, *t*, *r*) with confidence intervals, effect sizes, degrees of freedom and *P* value noted<br>*Give P values as exact values whenever suitable.*
☐	☒	For Bayesian analysis, information on the choice of priors and Markov chain Monte Carlo settings
☐	☒	For hierarchical and complex designs, identification of the appropriate level for tests and full reporting of outcomes
☐	☒	Estimates of effect sizes (e.g. Cohen's *d*, Pearson's *r*), indicating how they were calculated

Our web collection on statistics for biologists contains articles on many of the points above.

Software and code

Policy information about availability of computer code

Data collection	This study focused on the reanalysis of already published datasets: (1) Cell cycle SEC-SWATH-MS dataset (Heusel et al. 2020) , (2) mouse tissue SWATH-MS dataset (Williams et al. 2018), and (3) replicate HEK293 cell line measurements (Collins et al. 2017). All dat was downloaded from ProteomeXchange (http://proteomecentral.proteomexchange.org) via the PRIDE partner repository (see below for all accession codes). No additional software was used for data collection.
Data analysis	R version 3.6.2<br>Skyline version 4.1<br>CCprofiler (https://github.com/CCprofiler/CCprofiler) proteoformLocationMapping branch (doi:10.5281/zenodo.4762014)<br>Custom data anlysis scripts (https://github.com/ibludau/ProteoformAnanlysis) main branch (doi:10.5281/zenodo.4762047)<br>PeCorA (https://github.com/jessegmeyerlab/PeCorA) master branch<br>DAVID Bioinformatics Resources 6.8 (https://david.ncifcrf.gov/)

For manuscripts utilizing custom algorithms or software that are central to the research but not yet described in published literature, software must be made available to editors and reviewers. We strongly encourage code deposition in a community repository (e.g. GitHub). See the Nature Research guidelines for submitting code & software for further information.

Data

Policy information about availability of data

All manuscripts must include a data availability statement. This statement should provide the following information, where applicable:

- Accession codes, unique identifiers, or web links for publicly available datasets
- A list of figures that have associated raw data
- A description of any restrictions on data availability

All data presented in this study has been published before and is available on ProteomeXchange (http://proteomecentral.proteomexchange.org) via the PRIDE partner repository. The SEC-SWATH-MS data of HeLa cells in interphase and mitosis was published by Heusel et al. and is available via the identifier PXD010288. The mouse tissue SWATH-MS data was previously published by Williams et al. and is available via the identifier PXD005044. The data used to generate the in silico benchmark was previously published by Collins et al. and is available via the identifier PXD004886.

Field-specific reporting

Please select the one below that is the best fit for your research. If you are not sure, read the appropriate sections before making your selection.

[x] Life sciences [ ] Behavioural & social sciences [ ] Ecological, evolutionary & environmental sciences

For a reference copy of the document with all sections, see nature.com/documents/nr-reporting-summary-flat.pdf

Life sciences study design

All studies must disclose on these points even when the disclosure is negative.

Sample size	This study focused on the reanalysis of already published datasets: (1) Cell cycle SEC-SWATH-MS dataset (Heusel et al. 2020) with 2 conditions, 3 replicates and 65 fractions each, (2) SWATH-MS dataset of tissue samples from the mouse BXD genetic reference panel including 5 tissues measured from 8 BXD mice (Williams et al. 2018), and (3) 21 replicate measurements of the HEK293 cell line measured at site 2 of the multi-laboratory SWATH-MS study (Collins et al. 2017).
Data exclusions	The mitochondria enriched samples from the mouse tissue dataset were not included in the analysis. Only samples measured at site 2 of the multi-laboratory SWATH-MS study were used.
Replication	Results of the cell cycle SEC-SWATH-MS dataset (Heusel et al. 2020) are based on 3 replicates and 65 fractions for each condition. High reproducibility of the experimental workflow was confirmed by Heusel et al. A mean peptide profile across replicates was used for COPF anlaysis in this study.<br>The mouse tissue dataset includes 5 tissues measured from 8 BXD mice each (Williams et al. 2018). The tissue specific proteoform results presented in this study are based on this very heterogeneous background and can be expected to be reproducible also across other mouse stains.
Randomization	Randomization was not used for MS measurements in the previous studies in which all datasets were acquired. MS acquisitions were performed in an order specific to each respective study design to ensure optimal MS performance and comparability.
Blinding	Blinding was not relevant for MS measurements in the previous studies in which all datasets were acquired. Blinding was also not relevant for data analysis with COPF, because the purely correlation based analysis does not take study design into account.

Reporting for specific materials, systems and methods

We require information from authors about some types of materials, experimental systems and methods used in many studies. Here, indicate whether each material, system or method listed is relevant to your study. If you are not sure if a list item applies to your research, read the appropriate section before selecting a response.

Materials & experimental systems

n/a	Involved in the study	
x		Antibodies
	x	Eukaryotic cell lines
x		Palaeontology and archaeology
	x	Animals and other organisms
x		Human research participants
x		Clinical data
x		Dual use research of concern

Methods

n/a	Involved in the study	
x		ChIP-seq
x		Flow cytometry
x		MRI-based neuroimaging

Eukaryotic cell lines

Policy information about cell lines

Cell line source(s)	As original reports on the cell cycle SEC-SWATH-MS dataset (Heusel et al. 2020) and the multi-laboratory SWATH-MS study (Collins et al. 2017) .
Authentication	As original reports on the cell cycle SEC-SWATH-MS dataset (Heusel et al. 2020) and the multi-laboratory SWATH-MS study (Collins et al. 2017) .
Mycoplasma contamination	As original reports on the cell cycle SEC-SWATH-MS dataset (Heusel et al. 2020) and the multi-laboratory SWATH-MS study (Collins et al. 2017) .
Commonly misidentified lines (See ICLAC register)	As original reports on the cell cycle SEC-SWATH-MS dataset (Heusel et al. 2020) and the multi-laboratory SWATH-MS study (Collins et al. 2017) .

Animals and other organisms

Policy information about studies involving animals; ARRIVE guidelines recommended for reporting animal research

Laboratory animals	As original report on the SWATH-MS mouse tissue dataset (Williams et al. 2018).
Wild animals	As original report on the SWATH-MS mouse tissue dataset (Williams et al. 2018).
Field-collected samples	As original report on the SWATH-MS mouse tissue dataset (Williams et al. 2018).
Ethics oversight	As original report on the SWATH-MS mouse tissue dataset (Williams et al. 2018).

Note that full information on the approval of the study protocol must also be provided in the manuscript.
